# Indocyanine green fluorescence imaging-assisted laparoscopy resection of retroperitoneal tumors in children: case report and literature review

**DOI:** 10.3389/fped.2024.1374919

**Published:** 2024-06-05

**Authors:** Yuanyuan Luo, Hong Zhang, Qiang Wu, Yan Chen, Zhihua Ye, Ruiyu Liu, Chengwei Chai

**Affiliations:** ^1^ Department of Gastrointestinal Surgery, Guangzhou Women and Children’s Medical Center, Guangzhou Medical University, Guangzhou, China; ^2^ Guangdong Provincial Key Laboratory of Research in Structural Birth Defect Disease, Guangzhou Women and Children’s Medical Center, Guangzhou Medical University, Guangzhou, China; ^3^ Department of Urinary Surgery, Guangzhou Women and Children’s Medical Center, Guangzhou Medical University, Guangzhou, China

**Keywords:** retroperitoneal tumor, children, laparoscopy, indocyanine green, real-time intraoperative navigation

## Abstract

This study examined the applicability of indocyanine green (ICG) fluorescence imaging to assist the laparoscopic resection of retroperitoneal tumors in pediatric patients via an abdominal approach. Conducted prospectively at the Guangzhou Women and Children's Medical Center from May to September 2023, the research included three pediatric cases, for whom laparoscopic retroperitoneal tumor resections were performed utilizing ICG fluorescence imaging. In each case, ICG was intravenously administered (0.3 mg/kg) prior to surgery, enabling the visualization of vital vascular structures through real-time fluorescence imaging. The trocar's placement was guided by a “four-hole” technique from the healthy side in a 70-degree lateral decubitus position. The operations were accomplished successfully without any complications. Pathological analysis of the patients identified one case of Wilms tumor of the embryonal type, one ganglioneuroblastoma of the mature type without N-MYC gene amplification, and one mature cystic teratoma. The findings suggest that with careful patient selection and skilled surgical execution, the utilization of ICG fluorescence imaging in the laparoscopic resection of retroperitoneal tumors is both safe and effective in children. This approach significantly improves the visualization of critical blood vessels, thus enhancing surgical safety.

## 1 Introduction

Surgical resection remains the cornerstone for curatively treating solid retroperitoneal tumors in pediatric patients, where complete tumor removal is imperative for a favorable outcome. The complex anatomical placement of these tumors, the confined surgical field, and their tendency to involve adjacent major vessels heighten the risk of vascular injury during resection. Open surgery was traditionally the preferred method until 1996 when Yamamoto et al. introduced laparoscopic resection for pediatric neuroblastomas (1). With advancements in pediatric surgery, laparoscopy has increasingly become the method of choice, while more recently, robot-assisted techniques have also emerged (2), although their use in China is limited due to availability constraints. Laparoscopic resections are performed via a retroperitoneal or transperitoneal approach. The retroperitoneal route offers superior lesion visualization, minimal interference, and less bleeding but demands greater anatomical acumen and prolonged learning (3). The transperitoneal route provides a larger working area and a shorter learning curve, but comes with increased risks of major vascular injuries. Indocyanine green (ICG) fluorescence imaging is notable for its high sensitivity and non-radioactivity, and has been used in adult surgeries where it enables real-time vascular visualization. It has recently found use in pediatric applications, including biliary atresia, congenital biliary dilatation, hepatoblastoma, and congenital megacolon (4, 5). However, its efficacy in enhancing the safety of laparoscopic retroperitoneal resections in children remains unconfirmed. This study, through a retrospective review of three pediatric cases, investigated the feasibility and safety of utilizing ICG-assisted laparoscopy for retroperitoneal tumors to mitigate the risk of vascular injury.

## 2 Case presentation

The inclusion criteria were: (1) Preoperative imaging showing retroperitoneal tumors not crossing the midline with a maximum diameter of <10 cm; (2) Absence of tumor thrombus in the renal vein and inferior vena cava, and no distant metastasis; (3) Ethical approval from the institutional review board and parental consent obtained prior to surgery. Three cases meeting the predetermined inclusion criteria were thus included in this study, with detailed clinical information provided below.

### 2.1 Case 1

A 3-year-old female patient weighing 13.9 kg presented with hematuria. Preoperative liver and kidney function were normal, and there were no lung or other site metastases. The tumor originated from the left renal pelvis, measuring 6.5 × 6.5 × 6.1 cm.

### 2.2 Case 2

An 11-year-old female patient weighing 39 kg was incidentally found to have an abdominal mass during a visit for upper respiratory tract infection. Preoperative liver and kidney function were normal, and there were no liver, spleen, or other site metastases. The tumor originated from the right adrenal gland of the kidney, measuring 5.7 × 3.8 × 5.1 cm.

### 2.3 Case 3

A 5-year-old female patient weighing 15.4 kg was incidentally discovered to have an abdominal mass during a visit for pneumonia. Normal preoperative liver and kidney function were observed, with no evidence of liver, spleen, or other site metastases. The tumor originated from the left adrenal gland of the kidney, measuring 4.8 × 3.5 × 5.0 cm.

## 3 Diagnosis and treatment

### 3.1 Diagnostic assessment

All patients underwent preoperative assessment using CT angiography. All three cases were diagnosed as retroperitoneal tumors through this imaging examination (Table 1).

**Table 1:** The time for each stage of treatment.

	Admission time	CT examination time	Operation time	Discharge time
Case1	2023.5.15	2023.5.17	2023.5.23	2023.6.1
Case2	2023.5.22	2023.5.23	2023.5.26	2023.6.8
Case3	2023.9.13	2023.8.16	2023.9.18	2023.9.27

### 3.2 Therapeutic intervention

After excluding surgical contraindications and after obtaining informed consent from the patient's guardian in each case, all three cases underwent laparoscopic tumor resections. The surgical method involved the following process.

**Figure 1:**
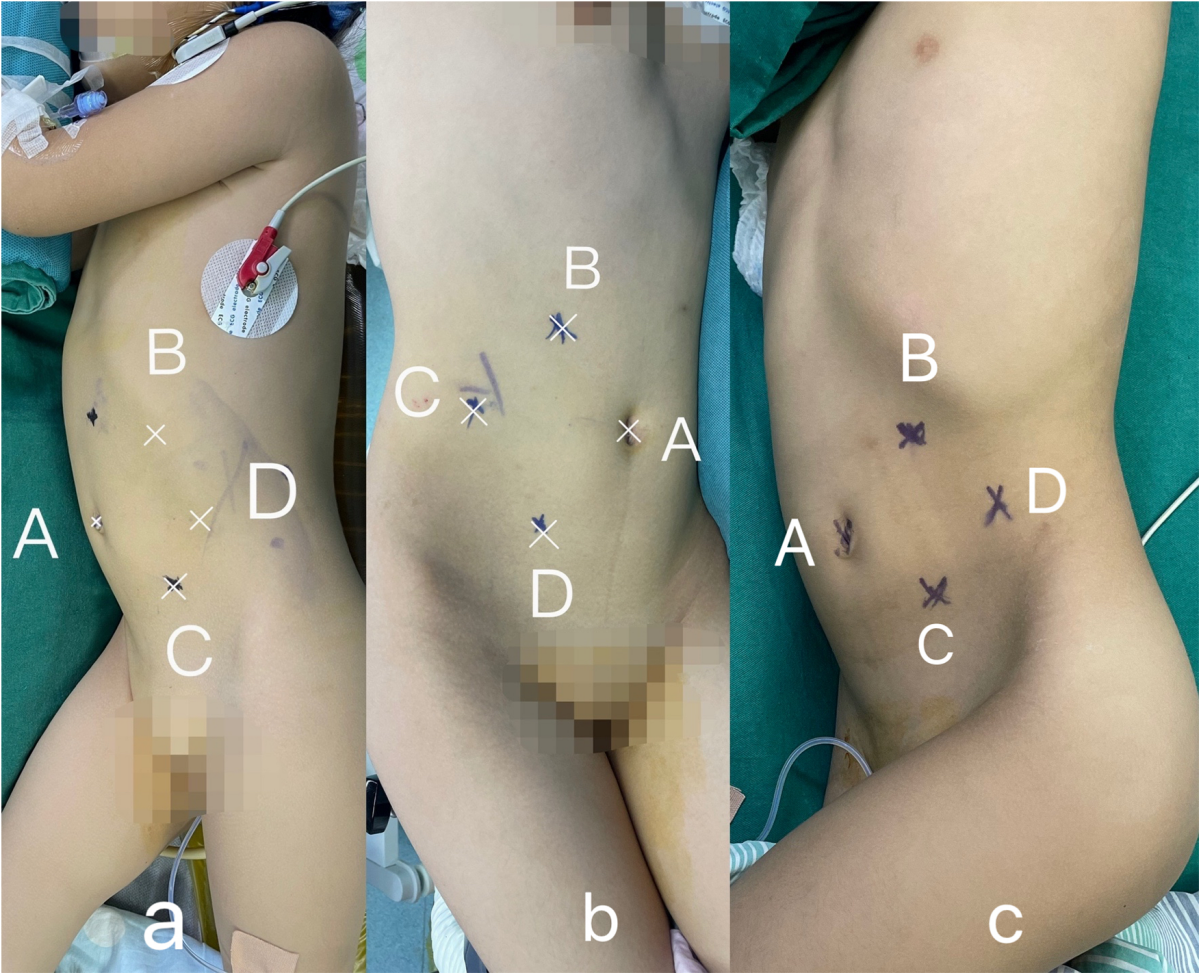
Patient positioning and trocar placement. (**A**) Case 1; (**B**) Case 2; (**C**) Case 3.

Patient positioning and trocar placement: Following general anesthesia, the patient was placed in a healthy-side 70° lateral decubitus position. The “four-port technique” was employed on the affected side. Here, the A port served as the umbilical observation port, while the B and C ports functioned as the primary working ports. These ports were positioned below the costal margin along the midclavicular line and at the level of the lateral edge of the rectus abdominis muscle, respectively. The D port was used as an auxiliary working port, and was located at the level of the anterior superior iliac spine along the midclavicular line (Figure 1).

Surgical procedure: (1) Initial puncture and entry through port A facilitated the introduction of a laparoscopic camera post-pneumoperitoneum creation. Subsequent exploration allowed for the placement of ports B and C under laparoscopic direction for insertion of the surgical instrument. For left-sided retroperitoneal tumors, the procedure commenced with an incision along the descending colon's margin, followed by the division of the gastrocolic ligament, splenic flexure, and splenorenal ligament, aiding in left hemicolon mobilization. The spleen and pancreas were elevated through an auxiliary port by the surgical assistant. The approach for right-sided tumors involved an incision along the ascending colon's outer margin, division of the hepatic flexure and hepatoduodenal ligament, and possibly a Kocher incision for mobilization of the duodenum and pancreatic head, with the assistant relocating the liver and duodenum as needed; (2) Priority was given to identifying and dissecting the major blood vessels. Retroperitoneal tumors, often entwined with major vessels and organs, necessitate careful instrument selection, including ultrasonic scalpels and electrocautery hooks, for effective adhesion dissection. Collaboration facilitates a gradual and considerate detachment of the tumor, reserving complex portions for later handling; (3) Intraoperatively, ICG (Dan Dong Medical Innovation Pharmaceutical Co., Ltd, China) was intravenously injected via the peripheral veins (0.3 mg/kg), and real-time fluorescence imaging was utilized to visualize the important vascular anatomical structures. This enabled a complete tumor resection guided by ICG fluorescence imaging (Zhuhai Deep Medical Technology Co., Ltd, China); (4) Following irrigation and immersion of the tumor bed, the resected specimen was placed in a specimen bag to prevent implantation metastasis.

### 3.3 Outcomes and follow-up

#### 3.3.1 Case 1

**Figure 2:**
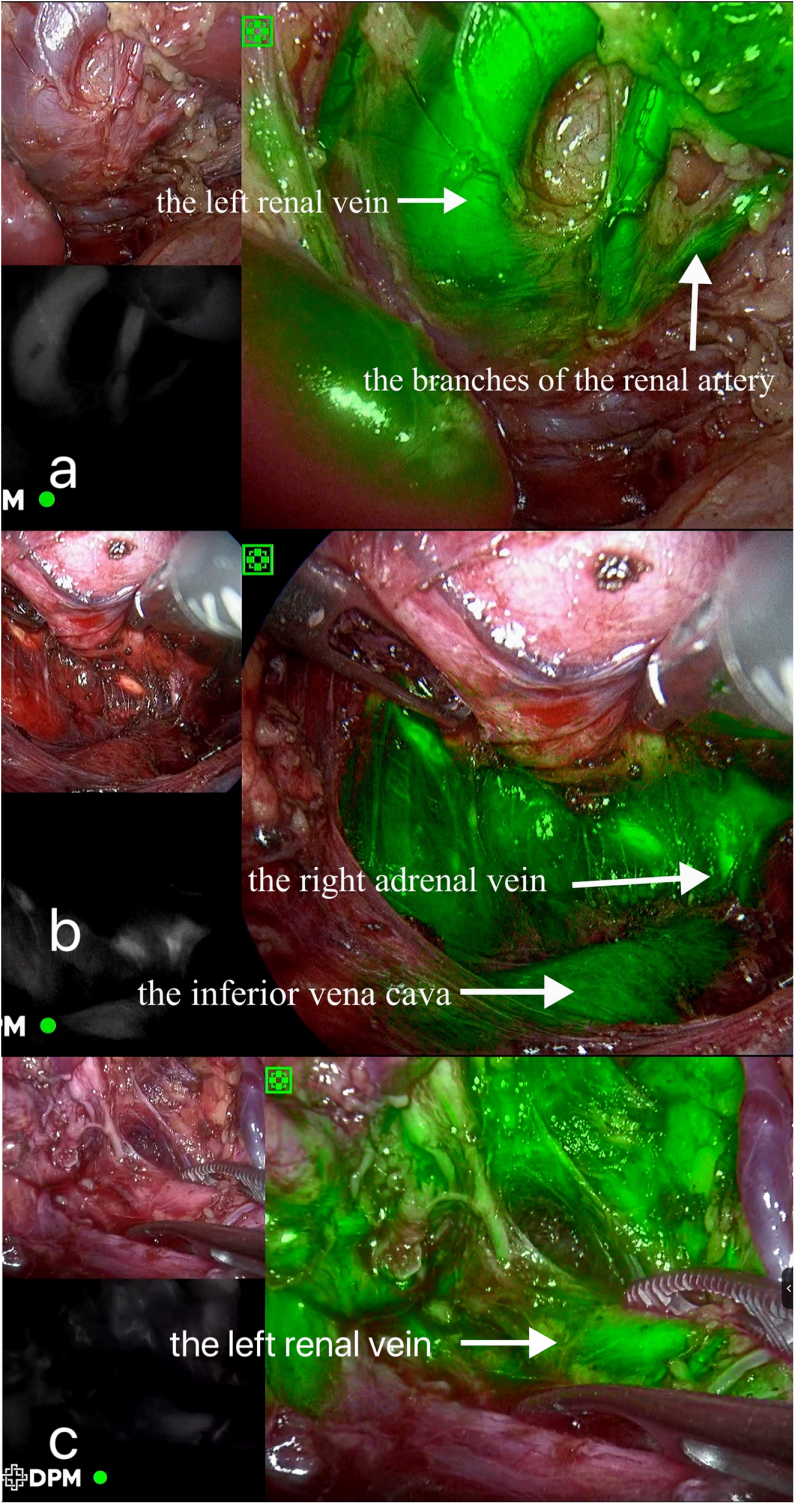
Intraoperative indocyanine green fluorescence imaging. (**A**) Case 1; (**B**) Case 2; (**C**) Case 3.

**Figure 3:**
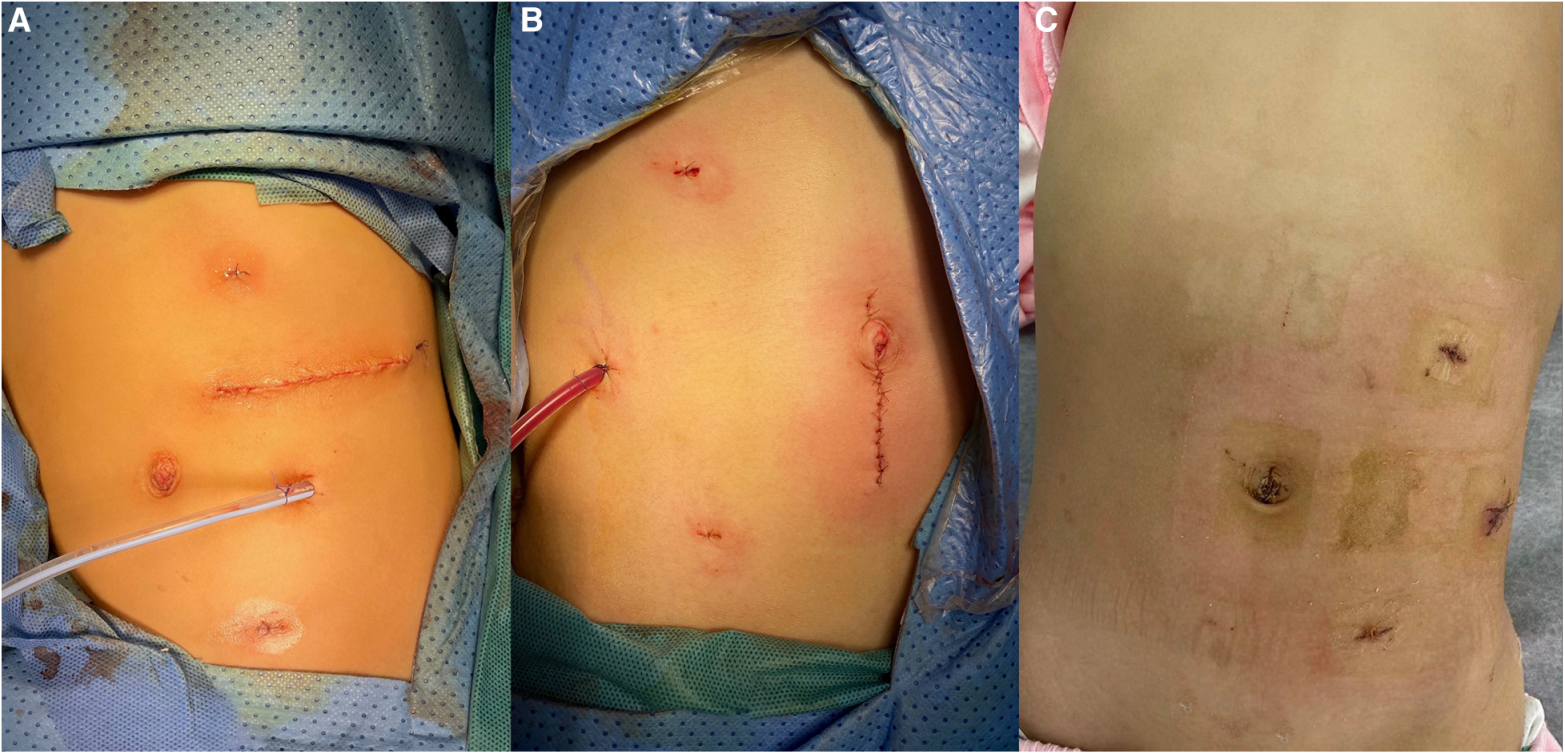
Postoperative incision and placement of the drainage tube. (**A**) Case 1, where the specimen was extracted through a left subcostal transverse incision; (**B**) Case 2, where the specimen was retrieved through a vertically extended trocar incision at the umbilicus; (**C**) Case 3, which involved a cystic tumor, where the cystic fluid was aspirated, and the cyst wall specimen was subsequently extracted through port D.

**Figure 4:**
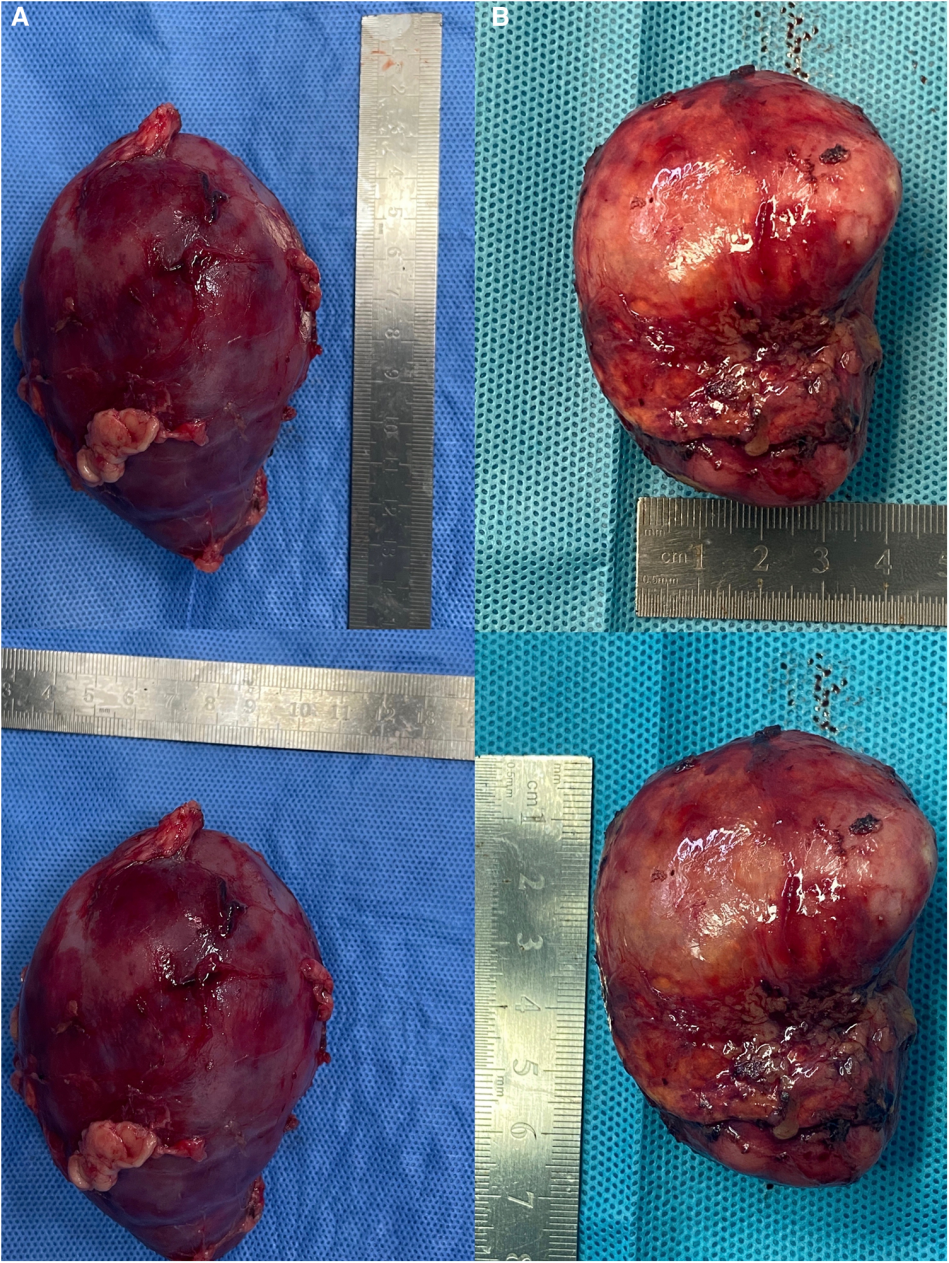
Tumor specimens. (**A**) Case 1, in which the dimensions of the specimen were measured as 11 × 7 × 5 cm; (**B**) Case 2, in which the dimensions of the specimen were measured as 6.5 × 5 × 4 cm.

Surgical findings: In this case, the left renal vein and superficial branches of the renal artery were visualized during surgery. The left renal vein appeared compressed and flattened due to the tumor pressure, with two adjacent branches representing the anterior trunk branches of the renal artery forming a “Y” shape (Figure 2A). Upon dissecting the tumor from the upper pole, the tumor capsule ruptured, resulting in an extrusion of the tumor tissue. The tumor specimen was removed through a transverse incision below the left rib margin (Figure 3A). The dimensions of the specimen were measured as 11 × 7 × 5 cm (Figure 4A).

Postoperative course: No surgery-related complications occurred, and the pathological examination confirmed the diagnosis of renal cell carcinoma, predominantly of the embryonal type. On the 6th day postoperatively, the patient was transferred to the oncology department for chemotherapy. The patient is currently undergoing scheduled chemotherapy.

#### 3.3.2 Case 2

Surgical findings: Visualization of the right renal vein, right adrenal vein, and the inferior vena cava was achieved, enabling dynamic observation of the right adrenal vein merging with the inferior vena cava (Figure 2B). Assisted by ICG fluorescence imaging, complete tumor excision was performed. The tumor specimen was extracted through a longitudinally extended trocar incision at the umbilical region (Figure 3B). The dimensions of the specimen measured 6.5 × 5 × 4 cm (Figure 4B).

Postoperative course: No surgery-related complications occurred. Pathological examination confirmed the diagnosis of a mature intermediate neuroblastoma originating from chromaffin cells, without N-MYC gene amplification. The patient was discharged on the 13th day postoperatively. One-month follow-up in the outpatient department revealed no clinical symptoms and no evidence of tumor recurrence.

#### 3.3.3 Case 3

Surgical findings: Visualization of the left renal vein, containing three branches that traversed the tumor, as well as a lymphatic duct, was successfully achieved (Figure 2C). With the assistance of ICG fluorescence imaging, complete excision of the tumor was performed. The cystic tumor specimen was suctioned and subsequently extracted through an extended trocar incision at port D (Figure 3C). The dimensions of the specimen were measured to be 5 × 5 × 4 cm.

Postoperative course: No surgery-related complications occurred. Pathological examination confirmed the diagnosis of a mature cystic terotoma. The patient was discharged on the 9th day postoperatively. One-month follow-up in the outpatient department revealed no clinical symptoms and no evidence of tumor recurrence.

## 4 Discussion

The retroperitoneal space is a common site for solid tumors in children. For benign and borderline tumors, complete resection of the lesion during surgery is considered the most effective method to reduce recurrence and malignant transformation. In cases of malignant tumors, primary tumor resection is an essential component of comprehensive treatment, as complete tumor excision plays a crucial role in reducing the recurrence rates and improving the overall survival in pediatric patients (6). However, the retroperitoneal space in children presents challenges due to its limited operative space, high surgical risks, and technical difficulties. Historically, open approaches have been favored to achieve optimal exposure, precise anatomical dissection, and effective hemostasis. In 1996, Yamamoto et al. first reported the use of laparoscopic techniques for the resection of neuroblastomas originating from the adrenal gland in children (1). Since then, laparoscopic approaches have gradually gained popularity for the surgical resection of retroperitoneal tumors in pediatric patients.

Laparoscopic surgery, albeit recognized for its enhanced visualization, minimally invasive nature, and expedited recovery, faces significant challenges related to the retroperitoneal space, owing to its deep location and restricted operative field. The complex nature of retroperitoneal tumors, characterized by their size, vascularity, potential malignancy, and proximity to major vessels and tissues, complicates complete resection and elevates the risk of vascular injury. Historically, tumors larger than 6 cm were deemed relatively unsuitable for laparoscopic removal (7), yet recent reports have documented the successful resection of even larger tumors, sparking an ongoing debate about the procedure (8, 9). Scholars contend that size alone should not preclude laparoscopic intervention, provided preoperative assessments ensure intact tumor capsules, the absence of midline crossing, clear demarcation, and no major vessel invasion (10–12). Our study, involving cases with tumors exceeding 6 cm, supports the view that the tumor size should not constitute an absolute contraindication. However, with malignant retroperitoneal tumors' propensity for rapid growth and a 9% risk of intraoperative rupture—a risk that escalates with tumors larger than 15 cm—meticulous patient selection becomes paramount (13). While we were able to successfully protect the surgical field and prevent extensive tumor spillage in Case 1, it is undeniable that s potential tumor rupture could adversely impact the tumor staging, treatment, and prognosis for pediatric patients. Our findings underscore the necessity of comprehensive preoperative imaging to gauge the tumor characteristics and assess the feasibility for laparoscopic intervention. Prototypical candidates should lack a history of abdominal surgeries, exhibit intact tumor capsules, show no major vascular involvement or adhesion, and the tumor should not traverse the midline. Laparoscopy appears relatively safe for tumors ≤ 10 cm in diameter, but for larger tumors, particularly over 10 cm, surgeons should proceed with caution, and consider open surgery if a complete and safe removal becomes doubtful.

The retroperitoneal and transperitoneal routes are primary surgical approaches for tackling tumors, with no significant differences in outcomes or complications reported between them (14). However, the transperitoneal route is typically favored by experienced laparoscopic surgeons due to its larger operative field and more familiar orientation. Although standardized trocar positioning remains varied, adherence to a diamond-shaped configuration is recommended for optimizing access and visibility. For adequate exposure of the tumor, extensive mobilization of the colonic ligaments is also necessary. In this study, a “four-port technique” was employed (Figure 1), taking advantage of gravity to expose the retroperitoneum effectively by allowing the displaced colon to shift away from the surgical field. This method positions the trocars in a diamond layout, facilitating precise manipulation by the primary surgeon and enabling assistance to be provided through an auxiliary trocar. The adoption of this technique can be advantageous for beginners because of its clarity and ease of use, despite the higher technical demands it imposes on the assistant, especially in pediatric laparoscopy. Due to children's smaller abdominal cavities, the assistant may need to manage both camera handling and assisting duties, a task that becomes more challenging during procedures on right-sided tumors, as it must be done using the left hand. It is thus crucial that such surgeries are undertaken by a cohesive surgical team that is well-versed in pediatric laparoscopic operations.

The transperitoneal approach, despite its shorter learning curve, faces challenges from abdominal organ and major blood vessel interference, complicating tumor exposure and elevating the risk of vascular injury. Retroperitoneal tumors' propensity to invade, encase, or adhere to adjacent vessels often results in displaced and structurally altered blood passages, significantly heightening the surgical dangers (15). A 2005 Swiss multicenter study noted a 0.09% incidence of major vascular injuries in 43,028 laparoscopic surgeries (16), underscoring the major vascular injuries' association with considerable blood loss, rapid bleeding, and complex repairs. These injuries can precipitate particularly severe outcomes, like hemorrhagic shock or even death, without proper management. Thus, maintaining the anatomical integrity of the midline vessels is crucial for ensuring safety in retroperitoneal surgeries. This area, posing risks to blood vessels, including the abdominal aorta and renal and adrenal vessels (17), is particularly susceptible to venous injuries due to the delicate, less elastic nature of vessels and the reduced tactile feedback of laparoscopic instruments. Consequently, reducing vascular harm and increasing laparoscopic safety in the retroperitoneal space is a research priority.

ICG is a tricarbocyanine dye known for its near-infrared absorption and emission, with characteristic fluorescence around 840 nm. Once intravenously administered, it swiftly binds to plasma proteins and circulates within the bloodstream, allowing for real-time vascular visualization using specific detection devices. The liver ultimately metabolizes ICG, facilitating its excretion via glutathione S-transferase pathways (18, 19). The use of ICG fluorescence imaging can be divided into anatomical and physiological categories, whereby it can aid in the visualization of anatomical structures and in revealing tissue status, respectively. This study harnessed the anatomical imaging capabilities of ICG to evaluate the perfusion of key abdominal vessels, thus enhancing intraoperative navigation with an aim to minimize vascular injury risks. For instance, in Case 1, ICG imaging from post-intravenous ICG injection outlined a compressed left renal vein and superficial renal artery branches due to tumor compression. Case 2 displayed indistinct tumor boundaries with the right adrenal gland and pressure on the inferior vena cava's medial side; subsequent ICG injection highlighted the right adrenal vein and inferior vena cava, offering a dynamic view of venous flow from the adrenal gland. Lastly, Case 3 showed the left renal vein adjacent to a tumor situated between the left adrenal gland and kidney, with ICG revealing three branches traversing the tumor. Across these three cases, ICG imaging underpinned the precise vessel localization and condition assessment, crucially reducing the risk of intraoperative vascular injury.

This study has some limitations to note. First, due to the limited number of reported studies on this technique, there is a scarcity of available experience to draw upon. Therefore, we adopted a conservative approach in establishing our inclusion criteria during the exploratory phase, resulting in a relatively small sample size. The generalization of our findings would necessitate larger sample sizes and multicenter prospective studies. Second, our experience with fluorescence imaging techniques for vascular localization is still developing. Currently, the dosage and timing of ICG administration primarily rely on the operator's experience and the existing literature. Therefore, further investigation is warranted to determine the optimal dosages, administration timing, and other related factors. Finally, future research should focus on exploring methods to achieve continuous and stable imaging and investigating the factors that may influence the administration of ICG.

In summary, the utilization of ICG fluorescence imaging-assisted laparoscopic retroperitoneal tumor resection in children has been demonstrated to be both safe and feasible. This technique combines the advantages of minimally invasive surgery with the operational experience derived from open surgery. Fluorescence imaging assisted by ICG facilitates intraoperative anatomical localization of the critical blood vessels and enables real-time navigation, thereby enhancing surgical safety. However, it is crucial to selectively choose the appropriate cases and ensure the involvement of a highly skilled surgical team with significant experience in laparoscopy.

## 5 Patient perspective

In the journey of treating a child's retroperitoneal tumor, the child's guardians typically experience significant apprehension regarding the forthcoming surgical intervention. In the presented cases, the decision to opt for an ICG fluorescence imaging-assisted laparoscopic approach came after thorough consultations and a comprehensive understanding of the potential benefits. The guardians' initial concerns were naturally centered around the safety and efficacy of this relatively novel technique, especially considering the delicate condition of their child.

Post-surgery, the guardians reported a profound sense of relief and satisfaction with the treatment outcome. They were particularly impressed by the minimally invasive nature of the procedure, which resulted in notably reduced postoperative discomfort and a quicker recovery period for their child. The use of ICG fluorescence imaging was recognized by the guardians as a pivotal factor in the surgery's success; it was reassuring for them to know that the surgical team had an enhanced visualization of the vital vascular structures throughout the procedure, thereby significantly mitigating the risk of potential complications.

The positive histopathological outcomes further solidified the guardians' confidence that they made the right decision for their child to undergo this sophisticated surgical technique. Witnessing their child's swift return to normalcy and health post-operation brought immense comfort to the families. Furthermore, the guardians expressed a deep appreciation toward the surgical and medical teams at the Guangzhou Women and Children's Medical Center for their exceptional care and expertise. The experience had not only restored their child's health but also instilled a sense of hope and trust in advanced medical interventions.

## Data Availability

The raw data supporting the conclusions of this article will be made available by the authors, without undue reservation.
